# Epidemiological profile of cutaneous leishmaniasis: Retrospective analysis of 7444 cases reported from 1999 to 2005 at Ouagadougou, Burkina Faso

**DOI:** 10.11604/pamj.2013.14.108.1140

**Published:** 2013-03-19

**Authors:** Sanata Bamba, Alphonse Gouba, Maxime Koiné Drabo, Désiré Nezien, Mamadou Bougoum, Tinga Robert Guiguemdé

**Affiliations:** 1 Superior Institute of Health Sciences, Laboratory of Parasitology-Mycology, Polytechnic University of Bobo-Dioulasso, Burkina Faso; 2 Unity of Training and Research Institute of Health Sciences, University of Ouagadougou, Burkina Faso; 3 Research Institute of Health Sciences, Bobo Dioulasso, Burkina Faso; 4 National Laboratory of Public Health, Ouagadougou, Burkina Faso

**Keywords:** Trend, cutaneous leishmaniasis, Epidemiology, neglected disease, Ouagadougou

## Abstract

This retrospective study was aimed to describe the trend of the cases and to determine the annual incidence rate of cutaneous leishmaniasis from 1999 to 2005 in the city of Ouagadougou. To achieve these objectives, a retrospective study was conducted. Data collection was conducted from January 1999 to December 2005. In total, 7444 cases of cutaneous leishmaniasis were recorded with an annual average of 1063.30 ± 270. 8 cases. The sex ratio M/F was 0.9. The average age was 22.8 ± 13.5 years. Patients more than 15 year-old accounted for 72.5%. A decrease in the cases of the disease was noted during the months of March, April, May, June, and December. The peak was recorded during the months of September and October. Over 7 years, the average incidence rate was 0.1% ± 0.04 but does not reflect the importance of this pathology. Thus, a prospective study was recommended.

## Introduction

Leishmaniasis is a disease caused by protozoan parasites of the *Leishmania* genus, transmitted to man by the bite of an insect, known as sand-fly [1]. The disease has different clinical forms, ranging from a skin ulcer, which can heal spontaneously, to the most severe form of leishmaniasis, the visceral form, which can lead to patient′s death when untreated [2]. The exposed population is 350 million people and the global prevalence is 12 million cases [2]. Several forms of the disease have been reported in the continents of Europe, Asia, Africa and the Americas [2–5]. Due to its frequency and lethality, mainly in untreated patients, it is currently among the six endemic diseases considered as priorities worldwide [2]. In Burkina Faso, the cutaneous leishmaniasis (CL) is an important public health problem. The first cases were reported in 1960 by Oddou [6]. Since 1996, there has been an increase in reporting cases of cutaneous leishmaniasis in the city of Ouagadougou [7–9]. *Leishmania major* is the causative agent of cutaneous leishmaniasis in Burkina Faso [7]. Traoré et al identified 1847 cases between 1996 and 1998 and mapped the distribution of the disease in the city of Ouagadougou [8]. Guiguemdé et al. assessed at 13% prevalence rate of HIV co-infection - cutaneous leishmaniasis in the city of Ouagadougou in 2002 [9]. From these studies, recommendations were made for effective strategies to control the disease [8, 9]. But what - about the epidemiological situation today? The purpose of this present article is to study the evolution of cutaneous leishmaniasis in the town of Ouagadougou from cases reported between January1999 and December 2005 in public health centers and community.

## Methods

The present study was a retrospective review of medical records of cutaneous leishmaniasis notified to curative consultations of 30 public health centers and community in Ouagadougou. Capital of Burkina Faso, with 1300000 inhabitants, Ouagadougou includes two university hospitals and a network of 108 health centers in 4 health districts [10]. The city has problems with the steep increase of its population, among which buildings anarchic and health consequences (sewage, garbage accumulation, lack of drinking water, proliferation of disease vectors, such as mosquitoes, sandflies). Data collection was conducted from January 1999 to December 2005. The following patient variables were analysed: sex, age and period of the consultation. Data were tabulated and analyzed through descriptive statistics in a Microsoft Excel spreadsheet, release 2003. The study was approved by the Research Ethics Committee of Centre Muraz in Burkina Faso.

## Results

**Figure 1:**
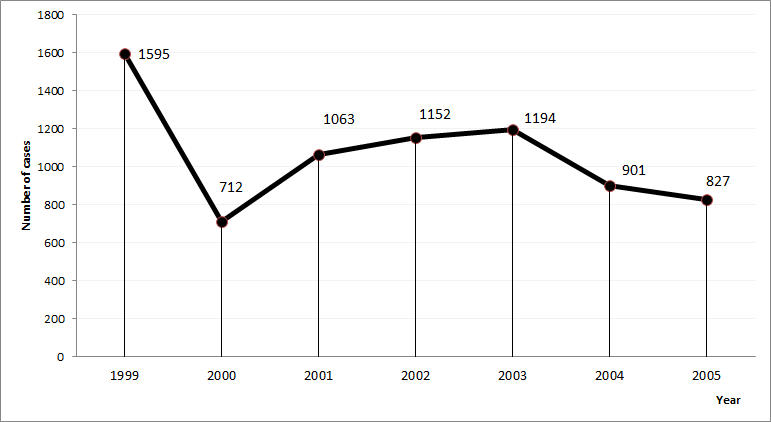
Distribution of 7444 cases of cutaneous leishmaniasis from 1999 to 2005 in Ouagadougou

**Figure 2:**
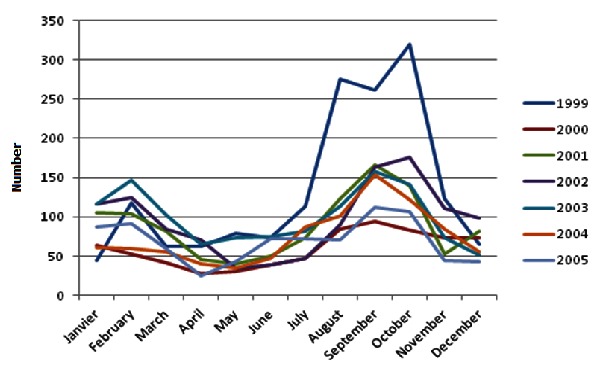
Representation of the comparative evolution of the monthly distribution of seven years.

During the period of January 1999 to December 2005, a total of 7444 cases of cutaneous leishmaniasis were notified. The annual average was 1063.3 ± 270.8 cases and the largest number was reported in 1999 (1595; 21.4% of all cases) (Figure 1). Regarding sex, female had the highest rate of CL infection, with 52. 9% (n=3938) followed by male with 47.1% (n=3506). The ratio sex was H / F = 0.9. The age of patients was noted for 2052 patients (whose information was available). It ranged from 1 year to 94 years and the average age. Patients aged less than or equal to 15 years accounted for 27.5% (564/2050) against 72.5% (1488/2050) for those older than 15 years (Table 1). The age groups most represented were those from 16 to 30 years (1063 cases or 51.80%) and 0 to 15 years (564 cases or 27.5%) (Table 1). According to the period, a decrease in cases of the disease was noted during the months of March, April, May, June and December. The peak was recorded in October for the years 1999 and 2002 and in September for the years 2000, 2001, 2003, 2004 and 2005 (Figure 2). During these seven years, the average incidence was 0.10% ± 0.04 (Table 2). The highest incidence was noted in 1999 with 0.19%.

**Table 1:** Representation by age group of 2052 cases

Age group	Number	Rate (%)
**0-15**	564	27.50
**16-30**	1063	51.80
**31-45**	292	14.20
**46-60**	96	4.70
**>60**	37	1.80
**Total**	2052	100

**Table 2:** Evolution of the number of cases and annual incidence of cutaneous leishmaniasis in Ouagadougou from 1999 to 2005.

Years	Population	Number of cases	Incidence (%)
**1999**	833761	1595	0,19
**2000**	863557	712	0,08
**2001**	894419	1062	0,11
**2002**	926384	1152	0,12
**2003**	1097018	1194	0,10
**2004**	1159340	901	0,07
**2005**	1225202	827	0,06

* Source: INSD available at http://www.insd.bf

## Discussion

It was noted an evolution in the sawtooth case. The decrease in the number of cases recorded from 1999 to 2000 could be linked to loss of records of consultations. Thus, it is found a small number of records of consultations compared to other years. That between 2004 and 2005 could be due to a regression of the disease in the city [11].

The year 1999 is when most cases are reported during this study. Traoré et al. were noted in a retrospective study from 1996 to 1998 that the disease increased in 1998 [8]. The results of this study confirm the progress of the cutaneous leishmaniasis.

The increase in cases notified between 2000 and 2004 was due to better diagnosis by the health personnel [5, 12, 13]. The peaks are noted in the month of October for the years 1999 and 2000 and in September for the years 2000, 2001, 2003, 2004 and 2005. Traoré et al. [8] in Ouagadougou and Keita et al. in Bamako, Mali [14] found a predominance of cases between July and September.

The monthly distribution showed low levels during the months of March (6.5%), April (4.5%), May (4.5%), June (5.3%) and December (6.2%). The high rates were recorded during the months of January (7.9%), February (9, 4%), July (6.9%), August (11.5%), September (14.8%) and October (14.6%). The peaks are noted in October for the years 1999 and 2000 and in September for the years 2000, 2001, 2003, 2004 and 2005. These results corroborate those obtained in Ouagadougou by Traoré et al [8] and those noted by Keita et al. in Bamako, Mali, which showed a predominance of cases between July and September [14].

The average annual incidence recorded was 0.10% ± 0.04. This rate does not seem to reflect the importance of this pathology. Because, it heals spontaneously after several months and does not disrupt daily life, many patients do not consult health centers for cutaneous leishmaniasis [2]. Thus, a prospective study would allow better assessment of the impact of this disease.

A sex ratio M/F of 0.9 is explained by a high frequentation of health centers by women (antenatal and infant consultations, etc.). The average age of 22.8 ± 13.5 years and the predominant age 16-30 years (51.8%) were found in Ouagadougou [8] and Morocco [15].

## Conclusion

Cutaneous leishmaniasis is a disease that has accompanied mankind since ancient times. There has been an increase in the number of cases in the last 20 years, as well as expansion to new geographic areas. Currently, the progression of the disease is a public health trouble and represents a challenge for health professionals. Epidemiological studies might help planning for effective strategies to control cutaneous leishmaniasis. This retrospective study aimed to describe the evolution of the number of cases of CL during 1999 to 2005 and to determine its annual incidence of cutaneous leishmaniasis in the city of Ouagadougou. During these seven years, the average incidence rate was 0.1% ± 0.04, but does not seem to reflect the real importance of this disease. All in all, a prospective study is recommended.
